# Human fitting of pediatric and infant continuous-flow total artificial heart: visual and virtual assessment

**DOI:** 10.3389/fcvm.2023.1193800

**Published:** 2023-07-17

**Authors:** Chihiro Miyagi, Munir Ahmad, Jamshid H. Karimov, Anthony R. Polakowski, Tara Karamlou, Malek Yaman, Kiyotaka Fukamachi, Hani K. Najm

**Affiliations:** ^1^ Department of Biomedical Engineering, Cleveland Clinic Lerner Research Institute, Cleveland, OH, United States; ^2^ Department of Thoracic and Cardiovascular Surgery, Cleveland Clinic, Cleveland, OH, United States; ^3^ Department of Biomedical Engineering, Cleveland Clinic Lerner College of Medicine of Case Western Reserve University, Cleveland, OH, United States; ^4^ Department of Pediatric Cardiology, Cleveland Clinic Children’s Hospital, Cleveland, OH, United States

**Keywords:** pediatric heart failure, congenital heart disease, mechanical circulatory support, pediatric total artificial heart, infant total artificial heart

## Abstract

**Background:**

This study aimed to determine the fit of two small-sized (pediatric and infant) continuous-flow total artificial heart pumps (CFTAHs) in congenital heart surgery patients.

**Methods:**

This study was approved by Cleveland Clinic Institutional Review Board. Pediatric cardiac surgery patients (*n* = 40) were evaluated for anatomical and virtual device fitting (3D-printed models of pediatric [P-CFTAH] and infant [I-CFTAH] models). The virtual sub-study consisted of analysis of preoperative thoracic radiographs and computed tomography (*n* = 3; 4.2, 5.3, and 10.2 kg) imaging data.

**Results:**

P-CFTAH pump fit in 21 out of 40 patients (fit group, 52.5%) but did not fit in 19 patients (non-fit group, 47.5%). I-CFTAH pump fit all of the 33 patients evaluated. There were critical differences due to dimensional variation (*p* < 0.0001) for the P-CFTAH, such as body weight (BW), height (Ht), and body surface area (BSA). The cutoff values were: BW: 5.71 kg, Ht: 59.0 cm, BSA: 0.31 m^2^. These cutoff values were additionally confirmed to be optimal by CT imaging.

**Conclusions:**

This study demonstrated the range of proper fit for the P-CFTAH and I-CFTAH in congenital heart disease patients. These data suggest the feasibility of both devices for fit in the small-patient population.

## 1. Introduction

In the pediatric heart failure (HF) population, the need for long-term support therapies such as the use of mechanical circulatory support (MCS) devices has become more prevalent in the last decade. The ventricular assist device (VAD), a representative MCS device, has yielded better outcomes recently (1–4) and it is reported that nearly one-third of pediatric patients receiving heart transplantation surgery are on VAD support (5). However, the donor hearts available for these children remain scarcer than those for adults; waitlist of infant mortality remains high (17%–30%) (6) compared to adults (7%) (7).

As for the recipient characteristics of the pediatric population undergoing heart transplantation, dilated cardiomyopathy (DCM) is the most common diagnosis globally; in North America, however, DCM and congenital heart disease (CHD) were equally prevalent as the diagnosis for heart transplantation recipients (both about 40%) (8). For this CHD population, both two-year survival post-device implantation and post-transplantation following VAD support are significantly worse compared with the DCM population (5). A probable explanation is the presence of anatomical and physiological challenges such as shunt-dependent circulations and/or single ventricle circulations, causing technical issues with a pump design that focuses only on biventricular circulation patients. Therefore, advanced-stage pediatric HF patients, especially CHD patients, might have benefited from replacement therapy, such as the total artificial heart (TAH) (9–12).

In particular, due to the specific developmental, anatomical, and morphological representations in the pediatric population (i.e., bilateral HF with single ventricular patients), the applications of currently available VADs are limited. In addition, due to the small size of the chest cavity, the applications and considerations are also limited to select patients. Therefore, more durable solutions are critical to address this clinical burden. Moreover, these patients need longer-term options even after the heart transplant, since the risk of chronic rejection or infection after a transplant is much higher.

Among viable clinical alternatives currently available, the only option as a pediatric TAH is the SynCardia 50 cc (SynCardia, Tucson, AZ). Although some space in the thoracic can be made after ventricular resection, the recommended patient size for this pump requires a body surface area (BSA) of 1.2–1.7 m^2^ (9, 10, 13), leaving a large number of pediatric patients ineligible due to their body size.

The pediatric continuous-flow TAH (P-CFTAH) is a pediatric-size TAH that is being developed by scaling down the adult CFTAH. Using the same algorithm as the adult CFTAH, the P-CFTAH produces self-balancing left and right circulations without electronic intervention, and is currently at the *in vivo* experimental stage of development (14). We also have conceptualized an even smaller pump designated as the infant CFTAH (I-CFTAH). Here we report the results of our initial fitting study of the P-CFTAH and I-CFTAH that were designed to confirm the feasibility of anatomical implantation, and to explore the appropriate range of patient sizes that would permit the use of replacement MCS devices.

## 2. Materials and methods

### 2.1. Study design

This study was approved by the Cleveland Clinic's Institutional Review Board (#17-1706). A written consent for participation was obtained prior to the surgeries. Mean body weight (BW), height (Ht), and BSA were 8.2 ± 6.2 kg, 68.7 ± 22.8 cm, and 0.39 ± 0.21 m^2^, respectively. The patients' diagnosis data are summarized in Supplementary Table S1.

### 2.2. Study inclusion criteria

The patients listed for surgery at the Cleveland Clinic Department of Thoracic and Cardiovascular Surgery from July 2019 to June 2021, with a confirmed CHD and a need for corrective surgery via a median sternotomy with pericardiotomy, were enrolled and included in this study after consent was obtained (*n *= 40, average age: 18.0 months, ranging from 5 days to 4 years). Both bi-ventricular and single-ventricular cases, in addition to palliative surgeries, were included in this study.

### 2.3. Study exclusion criteria

Patients over >30 kg, preterm infants, and those who were already on cardiopulmonary bypass support at the time of the fitting were excluded.

### 2.4. 3D-printed models of the P-CFTAH and I-CFTAH pumps

**Figure 1:**
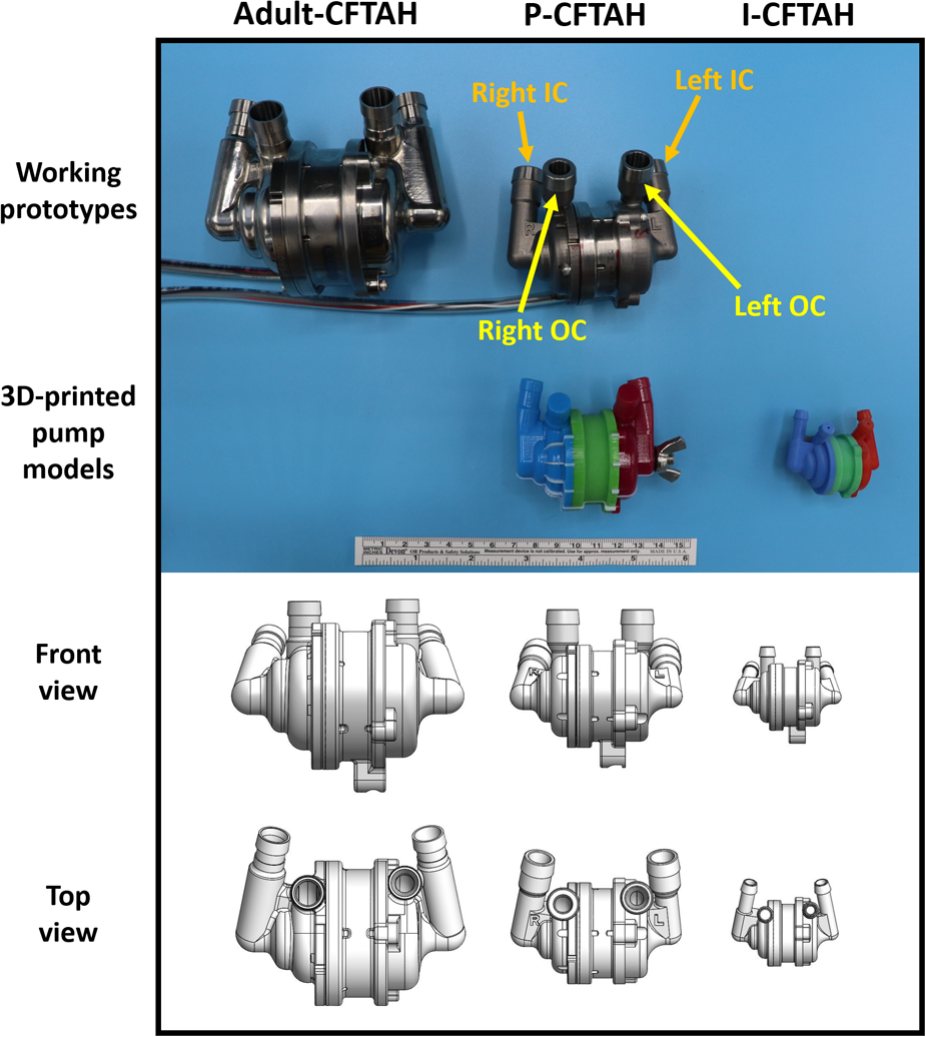
Size reference of three pumps (Adult CFTAH, P-CFTAH, and I-CFTAH). Top line: working pump prototypes of the adult CFTAH and P-CFTAH. Second line: 3D-printed models of the P-CFTAH and I-CFTAH used in intraoperative fitting studies. Third line: schematic illustrations of front view used for virtual fitting studies. Fourth line: schematic illustrations of top view used for virtual fitting studies. OC, outflow cannula; IC, inflow cannula.

The P-CFTAH pump was derived from the adult CFTAH by downsizing with a scale factor of 0.70, or approximately 1/3 of the total adult volume (Figure 1). The 3D-printed prototypes of the P-CFTAH (Supplementary Figures S1A–S1C) and I-CFTAH (Supplementary Figures S1D–1F) were developed for the intraoperative fittings. The 3D-printed model of the P-CFTAH follows the exact dimensions of the working prototype and has adjustable inlet and outlet conduits; as a result, the angulation and length can be adjusted to each patients' anatomies per case. The size of outlet conduit is not true to size, but they are attached to the pump for length and angle evaluation. Dimensions of the 3D-printed model of the I-CFTAH were downsized with a scale factor of 0.65 of the P-CFTAH; therefore, the inlet and outlet conduits are not adjustable and are used for size evaluation only. An actual working prototype of the I-CFTAH is presently under development.

### 2.5. Intraoperative fitting

**Figure 2:**
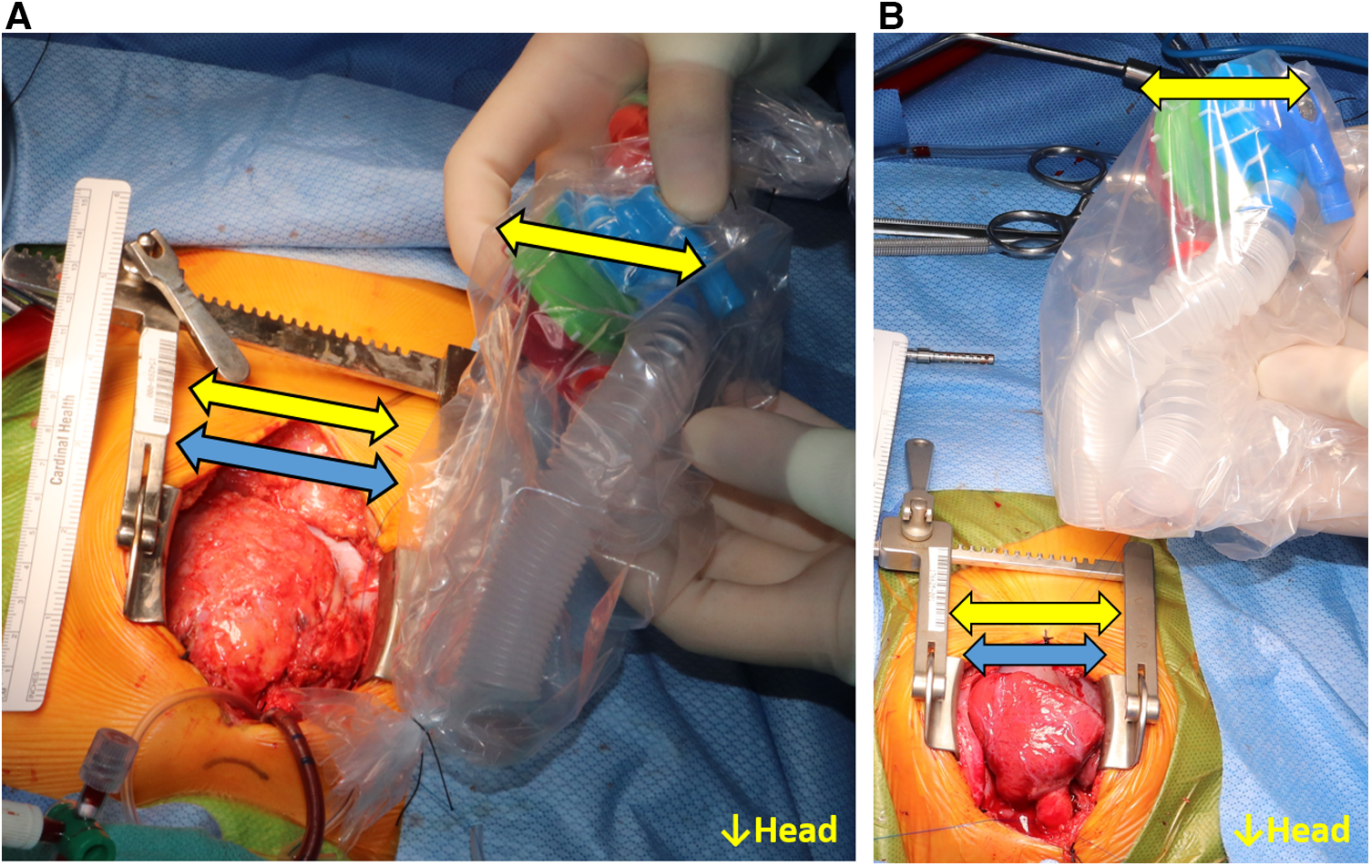
Intraoperative fittings for the P-CFTAH. (**A**) the patient categorized to the fit group, (**B**) the pump categorized to the non-fit group. Yellow arrow: size of the pump; blue arrow: size of the heart.

The 3D-printed models were sterilized through ethylene oxide gas sterilization. The sterilized models were used with a sterile plastic cover (iVAS Transducer Cover, CIVCO Medical Solutions, Kalona, IA), and the fitting procedure was performed from the first operator's position (patient right side). The size of the systemic ventricle was visually compared to the 3D-printed model, and the necessity of geometric adjustment to the inlet/outlet features of the P-CFTAH was also evaluated. If the model seemed to be of adequate size and implantable in the patient's thoracic cavity, the patient was assigned to the fit group (Figure 2A). If the ventricle seemed to be too small and not implantable for the pump, the patient was assigned to the non-fit group (Figure 2B). Since the concept of I-CFTAH was new, the first 7 cases were evaluated only for the P-CFTAH and in the remaining 33 studies, both P- and I-CFTAH were evaluated for fit.

### 2.6. Preoperative x-ray evaluation

For all patients enrolled in the fitting study (*n = 40*), their preoperative x-rays were evaluated besides the intraoperative fitting. From the front view (Supplementary Figure S2A): the thoracic width from left to right (A); the total heart size (B); the distance between the middle of vertebrae to apex (C); and the distance between the carina to apex (D) were measured. From the side view (Supplementary Figure S2B): the distance between sternum and middle of the vertebrae (E) was measured. Although a side view of x-rays is not always taken for all patients, those of 15 patients were available. All measurements were completed and recorded by one researcher, and the cardio-thoracic ratio (CTR) was also calculated as B/A.

### 2.7. Preoperative 3D-computed tomography image evaluation

Among the 40 patients, three had a contrast-enhanced computed tomography (CT) study performed prior to their intraoperative fitting study, which included a range of scans wide enough to evaluate the pump fitting. The CT datasets in the Digital Imaging and Communications in Medicine format were downloaded onto a computer workstation, and 3D on-screen models of the great vessels, rib cage, and heart without both ventricles were generated using Mimics Medical 22.0 software (Materialise, Leuven, Belgium) and exported in stereolithographic (STL) file format.

The anatomic models in STL format were then opened in the SOLIDWORKS application (Dassault Systèmes SOLIDWORKS Corporation, Waltham, MA), and the pump STL files, which were used to make the 3D-printed prototypes and were displayed with the anatomical models. The pump position was adjusted to determine an optimal pump fitting site (dependent on the patient's anatomy), and device interference with the surrounding tissues and chest walls was assessed from all directions. For all three anatomical models from the different size patients' CT data, both P-CFTAH and I-CFTAH pump data were applied and evaluated, respectively.

### 2.8. Statistical analysis

Data are presented as mean values with standard deviation (mean ± SD). Differences among quantitative parameters between the fitting and non-fitting groups were assessed using the Mann-Whitney *U* test (U). To investigate the cutoff value of each parameter, receiver operating characteristic (ROC) curves were constructed, and area under curve (AUC) for each ROC curve was evaluated. In all analyses, a value of *p* < 0.05 was considered statistically significant. Statistical analysis was performed using JMP Pro 14.2.0 software (SAS Institute Inc., Cary, NC).

## 3. Results

The P-CFTAH pump model fit 21 patients (*n* = 21; 52.5%), with the remaining patients (*n* = 19; 47.5%) classified to the non-fit group. By using the flexible conduit in mating length and alignment to the vessels, for all patients in the fit group, there was no need to adjust any angles of the inlet/outlet features of the pump. The mean age and BW of the fit group were 32.9 ± 32.3 months and 12.0 ± 6.4 kg, and those of the non-fit group were 1.47 ± 1.14 months and 3.94 ± 0.97 kg, respectively. Each group had significant differences for the BW (*p* < 0.0001), Ht (*p* < 0.0001), BSA (*p* < 0.0001), and thoracic and heart sizes (A–D: *p* < 0.0001, E: *p* < 0.05) (Table 1). There were no differences in the CTR. The fitting in the rest of the patients was not deemed feasible (*n *= 19) without expanding the space for physical device fit.

**Table 1:** Mean values of 40 patients for each parameter measured.

	Total (*n* = 40)	Fit group (*n* = 21)	Non-fit group (*n* = 19)	
Age (month)	18.0 ± 28.2	32.9 ± 32.3	1.5 ± 1.1	*p* < 0.0001
BW (kg)	8.2 ± 6.2	12.0 ± 6.4	3.9 ± 1.0	*p* < 0.0001
Ht (cm)	68.7 ± 22.8	83.8 ± 21.8	52.1 ± 6.0	*p* < 0.0001
BSA (m^2^)	0.39 ± 0.21	0.52 ± 0.21	0.23 ± 0.04	*p* < 0.0001
A (mm)	139.2 ± 30.8	160.6 ± 27.0	115.6 ± 11.4	*p* < 0.0001
B (mm)	81.8 ± 18.6	94.7 ± 16.2	67.5 ± 7.3	*p* < 0.0001
C (mm)	53.9 ± 15.2	62.3 ± 13.5	43.7 ± 10.3	*p* < 0.0001
D (mm)	75.8 ± 19.5	87.8 ± 18.0	61.2 ± 9.1	*p* < 0.0001
E (mm)	79.0 ± 14.6	82.1 ± 15.5	69.6 ± 4.7	*p* < 0.05
CTR (B/A)	0.59 ± 0.07	0.59 ± 0.07	0.59 ± 0.07	*p* = 0.89

BW, body weight; Ht, height; BSA, body surface area; CTR, cardio-thoracic ratio; A, the thoracic width from left to right; B, the total heart size; C, the distance between the middle of vertebrae to apex; D, the distance from the carina to the apex; E, the distance between the sternum and middle of the vertebrae in side view.

The cutoff values of each parameter obtained from the ROC curves are shown in Supplementary Table S2. Those of BW, Ht, and BSA were calculated as 5.7 kg, 59 cm, and 0.31 m^2^, with high AUCs of >0.95. These values are all relatively equivalent to the average size of 3–4 month-old children, and the BSA is much smaller than the minimum size reported by the SynCardia 50 cc case (0.9–1.1 m^2^) (9). One of the ROC curves (BSA) is shown in Supplementary Figure S3. As for the parameters obtained from the x-rays, A, B, and D also had clear cut-off values, with a high of AUC > 0.95. The distance between the sternum and middle of the vertebrae (E, *n* = 15) had a relatively low AUC (0.820) compared with other values, but was still high enough to make the cut-off line useful.

With regards to the I-CFTAH model, 33 out of 40 patients (range: 3.0–17.2 kg, mean: 7.10 ± 4.45 kg) underwent the intraoperative fitting evaluation following that of P-CFTAH. All showed an optimal anatomical fitting in the size of the chest and angles of the great vessels.

**Figure 3:**
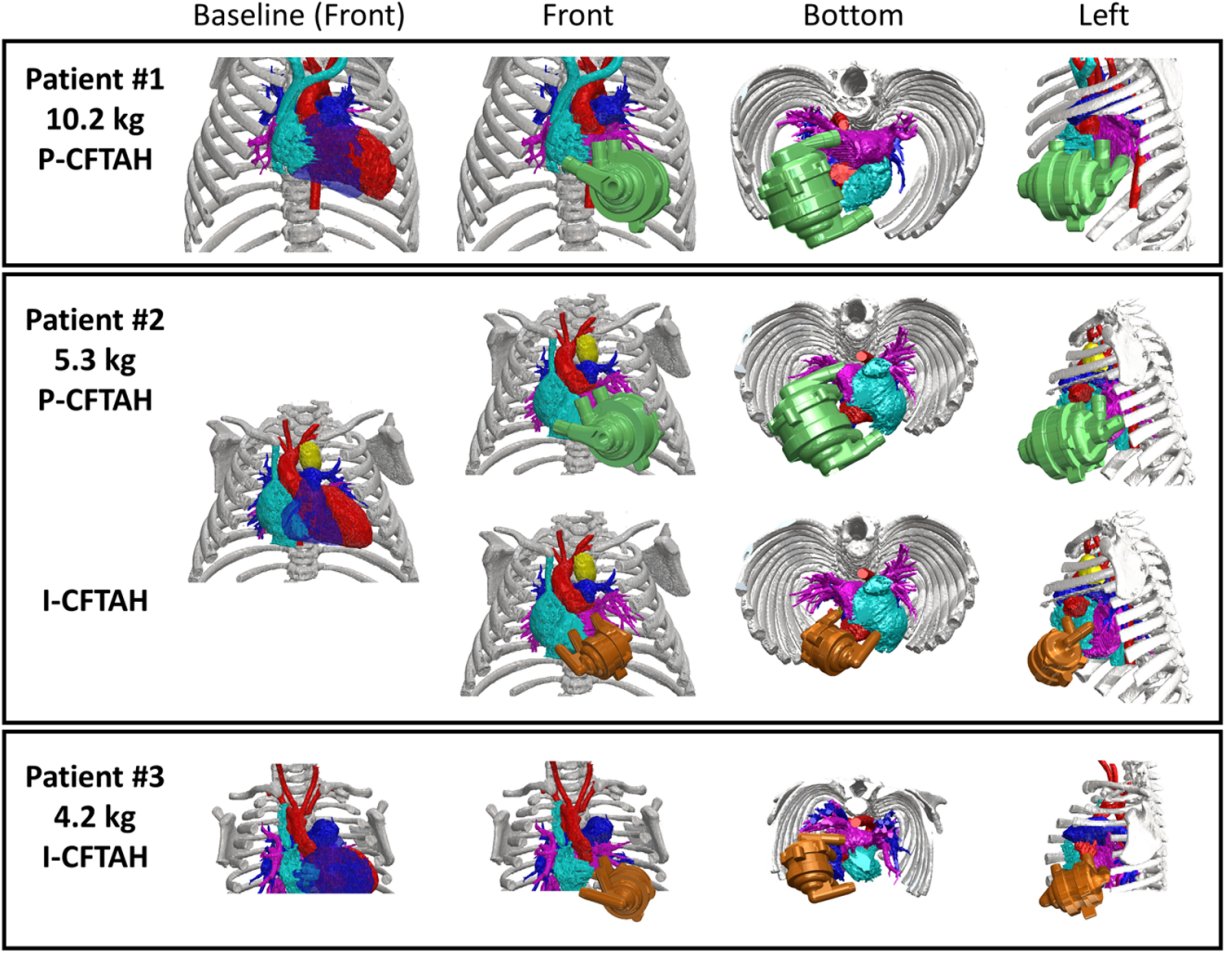
Combined views of three patients’ 3D-computed tomography images with and without P-/I-CFTAH pumps.

From the three preoperative 3D CT images, the combined views of the thoracic and the pump were obtained; the front, bottom, and left-side views are shown and compared in Figure 3. Patient #1 (10.2 kg, 16 months old) showed a proper fit for the P-CFTAH pump. Since this patient underwent heart transplantation, we were able to compare the pump prototype and the ventricles directly after resection of the ventricles. The images of patient #2 (5.3 kg, 4 months old) demonstrate how both the P-CFTAH and I-CFTAH pumps would be seen in the thoracic. According to the cutoff line of the BW obtained by the intraoperative fittings, the size of 5.3 kg is just below the line. Although the P-CFTAH in patient #2 looks much tighter than that in patient #1, it is still considered to be implantable with resection of the ventricles, and the intraoperative evaluation had actually assigned patient #2 to the fit group. Combined with the visual and virtual fitting, the cutoff line of 5.7 kg is considered to be probable and reasonable. Since the I-CFTAH model also showed an adequate fit in patient #2, this size patient (around 5.3–5.7 kg) could be a candidate for either pump. The 3D CT images of patient #3 (4.2 kg, 2 month-old) lack detail of the lowest part of the thoracic, including the diaphragm, but it is readily apparent that the I-CFTAH fits properly in this patient's chest cavity because size of the I-CFTAH is smaller than this patient's ventricles (Figure 3, bottom row).

## 4. Discussion

Children under 18 years old on the wait list for heart transplantation are known to experience some of the poorest mortality rates when compared to all other solid organ recipients in the United States (9, 15, 16). The outcomes of end-stage HF in the pediatric population have significantly improved recently due to the introduction of VADs (15, 17). When looking at their survival by age, however, the outcomes of the youngest group (age < 1year) have been the lowest. In the latest annual report of the Pediatric Interagency Registry for Mechanical Circulatory Support (Pedimacs), a strong appeal is made for better options for this population (18). This is largely because implantable continuous-flow VADs (IC-VADs), which have yielded superior results when compared to pulsatile VADs, are currently not available for infants and small children (19), and only 10% of IC-VADs were implanted in children under 20 kg (18).

As a result, the representative pulsatile VAD, the Berlin Heart EXCOR pediatric VAD (Berlin Heart GmbH, Berlin, Germany), tends to be used in younger, smaller patients with CHD. The Berlin Heart extracorporeal configuration with large-bore silicone cannulas, can carry blood across the abdominal wall and has less risk of lacking space in thoracic. However, the cannulas are prone to complications such as infection, cardiac tissue ischemia, and wound-healing problems in comparison with the smaller drivelines of implantable VADs. However, there are serious challenges to supporting patients with CHD with EXCOR VADs only, due to the complicated physiology, hemodynamics, and previous surgical interventions of CHD patients, in addition to the required hospital stay for the whole period while being treated with this device (2).

To resolve this critical deficiency, TAHs are getting more attention, since they enable biventricular support with a smaller size than VADs regardless of single- or bi-ventricle circulations. Especially in patients suffering from Fontan failure hemodynamics, VADs provide only systemic support that sometimes fails to benefit patients; thus, TAHs should be in consideration for improving hepatic congestion, renal insufficiency, or protein-losing enteropathy (17). However, only one TAH device, the SynCardia 50 cc, is currently approved by the Food and Drug Administration for pediatric use and even this cannot be considered for infants or small children with a BSA < 1.2 m^2^. It is an urgent goal to develop a novel smaller TAH for this population. The P-CFTAH was developed with this understanding, and the main aim is to support children < 1 year old and, more specifically, patient groups under 10 kg.

The adult CFTAH is a double-ended centrifugal pump with single rotor. The device is a valveless, continuous-flow pump, capable of automatic self-regulation to balance the systemic and pulmonary circulation passively, depending on the preload (left and right atrial pressures) (20). To address the clinical needs of smaller patients, the core device design transfer to a smaller-sized blood pump has been proposed. All the critical CFTAH features, including self-regulation, were incorporated into the P-CFTAH design; therefore, this pediatric device is also fully capable of balancing systemic and pulmonary flows. For patients with CHD, one of the biggest advantages of this self-balancing concept is that it can compensate for flow differences between left and right circulations due to bronchial shunts, which are commonly seen in CHD patients, and the shunt flow in patients requiring a TAH is said to be up to one-third of the systemic output (21).

The P-CFTAH pump has undergone a series of acute *in vivo* studies with lambs (∼30 kg), which confirmed that the prototype design has met the proposed performance parameters, including self-regulation and pulse modulation (14). The next step is to determine the feasibility and fit of this downsized device through intrathoracic human fitting studies, since the chest morphology and size of quadrupedal animals can differ largely from those of humans.

A similar fitting process had been conducted for the adult CFTAH, combining intraoperative and virtual fitting studies with CT scans (22), but for pediatric CHD patients, the number of available preoperative CT images with contrast was extremely small compared to those of adults. For evaluation, we accordingly attempted to use the x-ray images that are routinely taken preoperatively for most pediatric patients before any cardiac procedures. Combined with the intraoperative evaluation, the thoracic dimensions and cardiac sizes obtained from x-rays showed clear cut-off values with a large AUC for the differentiation between fit and non-fit; therefore, a preoperative x-ray is useful for evaluating whether the P-CFTAH is implantable.

Data accuracy of these cut-off values were effectively confirmed, even with a limited number of 3D-CT images. The technique of creating and combining on-screen images through generating 3D-reconstructions of complex anatomy and medical devices has been widely applied in diagnosis, patient education, and operative planning for complicated surgical approaches (23–25). Other TAH development processes have utilized a similar method for evaluation of fitting (26–28); so far, our lab has also had several experiences of virtual fittings with other pumps, using the Mimic software (29, 30). Size and anatomical fitting (i.e., distances and angles to the great arteries and both atriums) can be evaluated through 3D-CT fitting. Although the inlet/outlet angles of the P-CFTAH prototype are adjustable, the default angles were confirmed to be suitable for all fitting group patients and there was no need to adjust them. Adjustable diameters, angles, and lengths of the pump conduits at distal anastomosis sites will give much more flexibility in mating alignment to various native vessels and atria.

There are several limitations in this study. The first is that the visual intraoperative fitting could be subjective, and thus cannot be totally free of bias. Also, in this study, we could not put the device model into the chest directly due to Institutional Review Board regulations that prevent any possible infections by the study. The evaluation was done with eye measurements and by consensus, and although the method of comparing the chest and the pump was stylized as well as possible, there is still room for improvement in order to be more objective.

The second limitation is that the number of patients around the cut-off value of 5 kg was limited. More cases representing this BW are required to establish a more closely defined cut-off value; in some cases, a patient over the proposed cut-off line was placed in the non-fit group, and a smaller patient under the cut-off line placed in the fit group. Also, our previous study that compared the vertebra-to-sternum distances (the same parameter as E in the Supplemental Figure 2B) to BSA (31), showed that the P-CFTAH could fit in patients with a BSA ≥ 0.3 m^2^ (almost the same result as the current study), and that the parameter E was a good indicator for fitting. However, in this study, the AUC of parameter E was much less than other parameters. This can be explained by the fact that there are larger variations in the absolute values for vertebra-to-sternum distances than the not-CHD pediatric population due to the influence of previous surgeries for the CHD population. Recruitment of more CHD patients, ideally before VAD implantation or transplantation, is necessary. Since taking a CT scan with contrast before the surgery is not always a common strategy for most of the CHD population, related to this second limitation, we were only able to obtain three 3D-CT images from among the 40 patients. For the remaining patients, most of the imaging have been done by magnetic resonance imaging (MRI) if needed, but MRI has limited quality in resolution for fitting studies, and thus we decided not to use them.

The third limitation is that not all CHD patients require TAH implantation or transplantation, resulting in a discrepancy between the characteristics of the patient collection in this study when compared to the actual target population for the P-CFTAH/I-CFTAH pump. Among the 40 patients, simple CHD cases such as atrial septum defect patients are included, even though unlikely to experience severe HF, and only two DCM patients were included. However, these CHD patients' data were still useful in understanding the relationship between the actual heart size at surgery and the size shown in x-ray/CT, and we succeeded in confirming the reliability of x-ray measurements from them.

A final limitation is that this is merely a virtual or visual fitting. Careful evaluation of fit is still required at an actual implant surgery in the future. Also, even with careful preoperative evaluations, a risk of non-fit cannot be zero, so having some back-up plans, such as extracorporeal placement of a device would be necessary. Furthermore, since even the P-CFTAH pump cannot be used in patients smaller than 5 kg, the development of an actual working I-CFTAH prototype is needed as quickly as possible. This study's aim is only for size comparison. We did not intend to evaluate the sufficiency of cardiac output created by the pumps at this moment, but upcoming study will reveal these in near future.

## 5. Conclusion

This study demonstrated that the range of optimal dimensions for the P-CFTAH and I-CFTAH were feasible in pediatric CHD patients, including those who were under 10 kg at the time of evaluation. These results suggest the potential use of MCS devices is viable in small-sized pediatric HF patients. As a preoperative evaluation for fit, the BW, Ht BSA, and preoperative thoracic and cardiac dimensions obtained from thoracic radiography images were important parameters as a cutoff line for P-CFTAH and I-CFTAH implantation.

## Data Availability

The raw data supporting the conclusions of this article will be made available by the authors, without undue reservation.
