# Supplementary material for: Human fitting of pediatric and infant continuous-flow total artificial heart: visual and virtual assessment
Source: Front Cardiovasc Med. 2023 Jul 17;10:1193800. doi: 10.3389/fcvm.2023.1193800 (PMC10387526; doi:10.3389/fcvm.2023.1193800)
Supplement: Supplementary file 1 [file Datasheet1.docx]

**Supplementary Material**

**Supplemental Figure 1**:


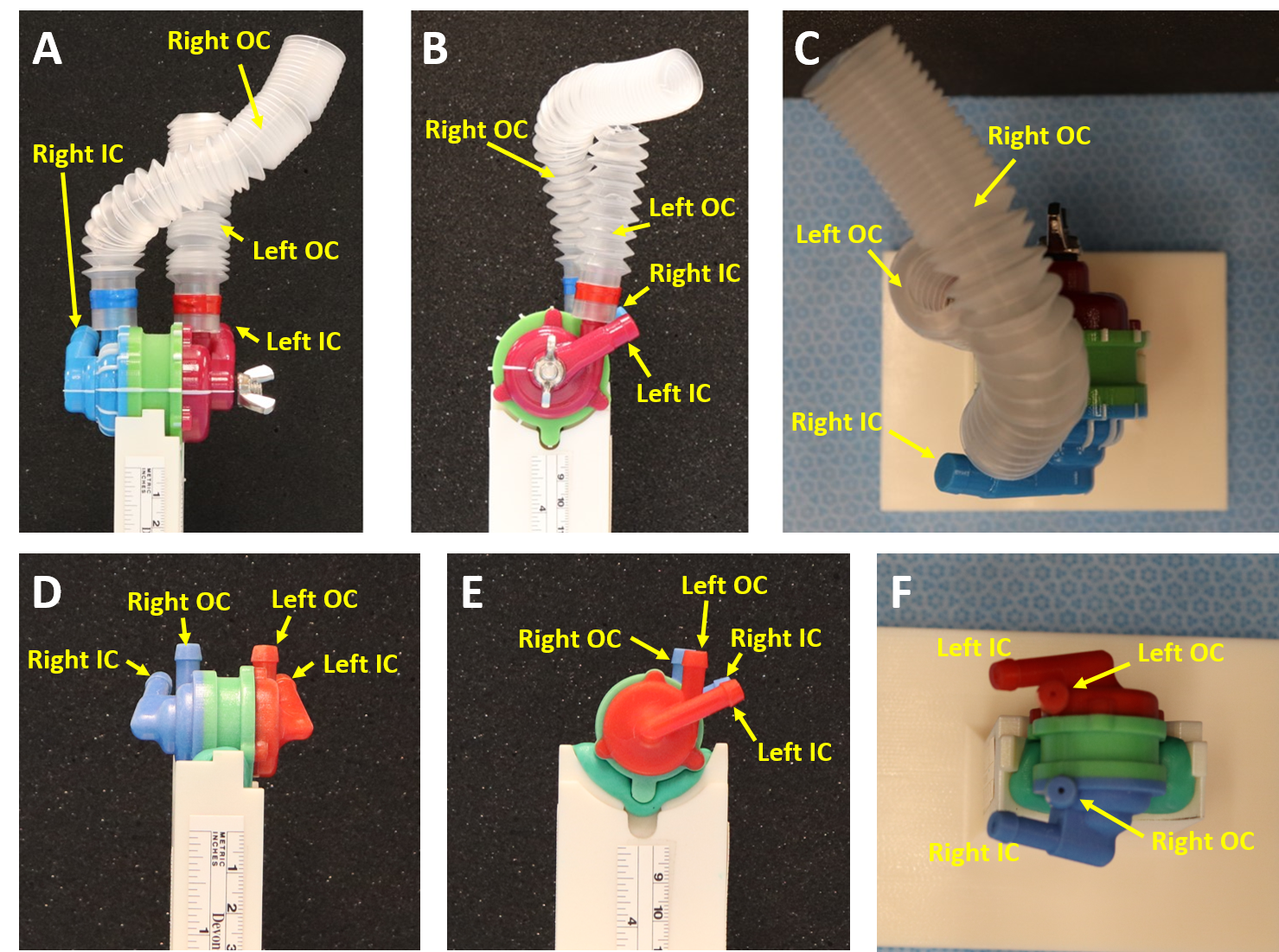


3D-printed P-CFTAH and I-CFTAH models. (**A**) front view, (**B**) side view, and (**C**) top view of the P-CFTAH. (**D**) front view, (**E**) side view, and (**F**): top view of the I-CFTAH. OC: outflow cannula, IC: inflow cannula

**Supplemental Figure 2**:


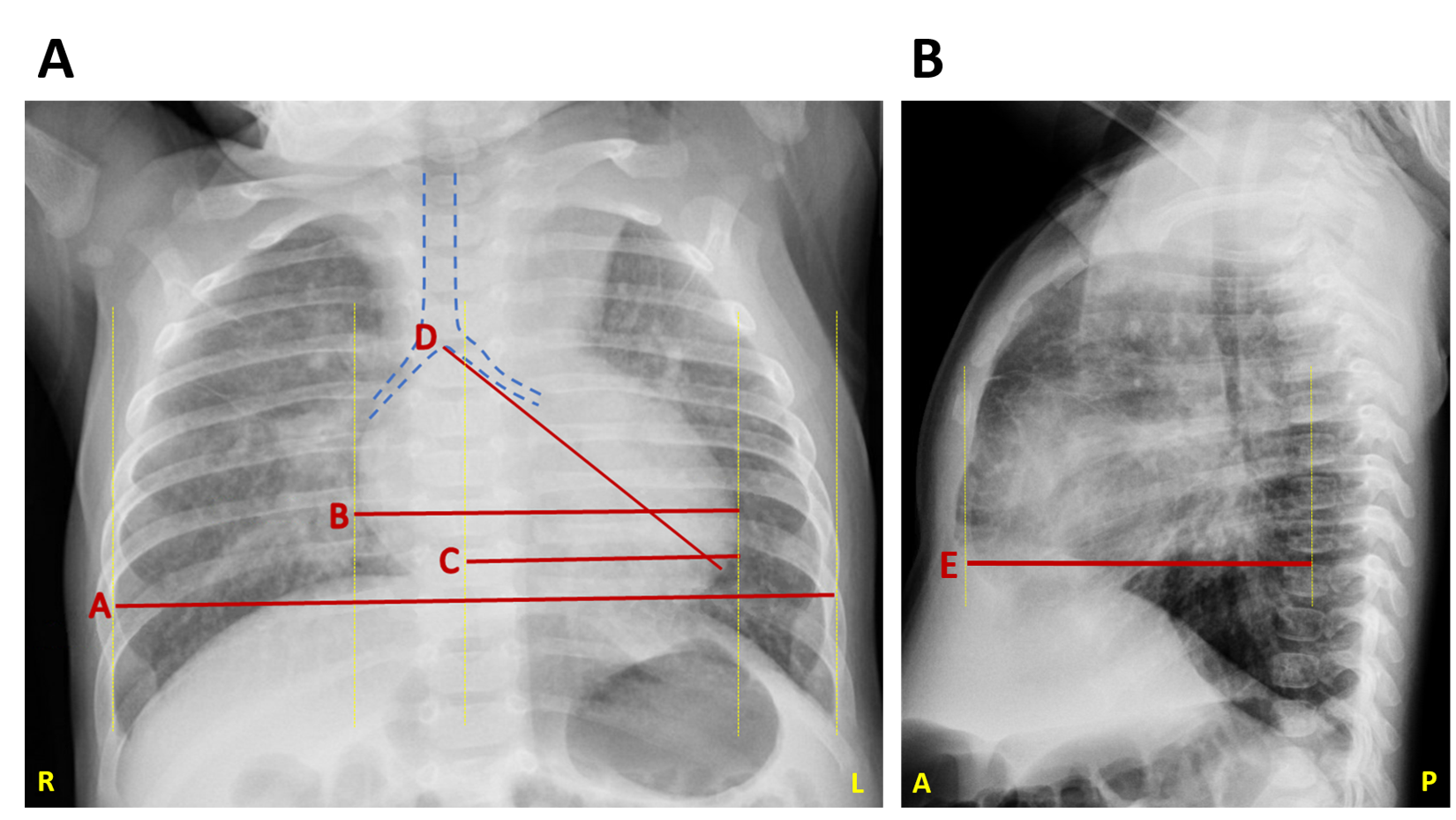


Measurement parameters of a preoperative X-ray. (**A**): the thoracic width from left to right, (**B**) the total heart size, (**C**) the distance between the middle of vertebrae to apex, and (**D**) the distance from the carina to the apex. (**E**) the distance between the sternum and middle of the vertebrae in side view. R: right, L: left, A: anterior, P: posterior

**Supplemental Figure 3**:


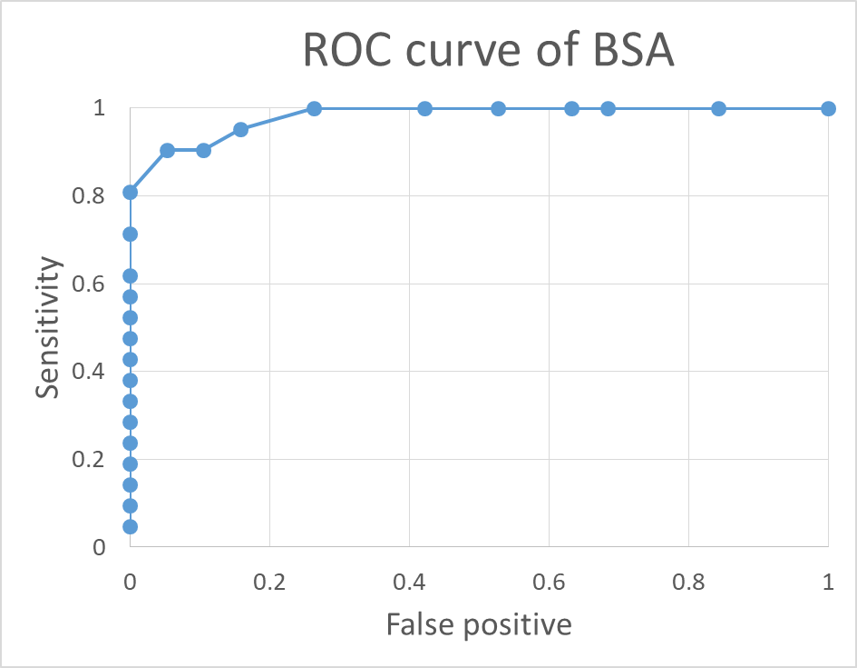


A receiver operating characteristic (ROC) curve of body surface area (BSA). The area under the curve is 0.9812, and the cut-off value is 0.31 m^2^, with a sensitivity of 0.9048 and a specificity of 0.9474.

| Patient # | Diagnosis | Procedures | Body weight (kg) |
| --- | --- | --- | --- |
| 1 | Ischemic cardiomyopathy | Berlin EXCOR implantation | 3.3 |
| 2 | ccTGA | Double switch | 30.0 |
| 3 | CoA, s/p CAVC defect repair | CoA repair | 4.56 |
| 4 | s/p Repair of ALCAPA | Heart transplantation | 10.2 |
| 5 | Cardiomyopathy | MV repair | 16.6 |
| 6 | VSD, PFO, PDA | VSD/PFO closure, PDA ligation | 5.1 |
| 7 | AP window, PFO | AP window repair | 4.8 |
| 8 | PAPVR, ASD, s/p TAPVR repair | PAPVR repair, ASD closure | 12.5 |
| 9 | VSD | VSD closure | 23.8 |
| 10 | Shone's complex | RV-PA conduit, MV repair | 10.8 |
| 11 | AS, MR s/p AVP | Ross procedure, MV repair | 5.64 |
| 12 | DCM, severe MR | Berlin Heart implantation | 8.9 |
| 13 | TV/RV hypoplasia, ASD, VSD | Berlin Heart implantation | 13.4 |
| 14 | MA, DILV, L-TGA, Dextrocardia | PAB | 3.09 |
| 15 | VSD, PFO | VSD closure | 4.8 |
| 16 | HLHS (MS/AA) | Norwood procedure | 3.88 |
| 17 | TOF | BT shunt | 3.21 |
| 18 | Truncus arteriosus | Truncal valve repair | 7.12 |
| 19 | HLHS (MS/AA) s/p Norwood, BDG | redo TCPC | 15.0 |
| 20 | AVSD | AVV repair | 3.15 |
| 21 | HLHS | Repair SV, TV valvectomy | 2.94 |
| 22 | VSD, PS | VSD closure, PS release | 5.71 |
| 23 | sub-AS (BAV), MR | sub AS release, MVP | 13.8 |
| 24 | ASD | ASD closure | 17.2 |
| 25 | AVSD, CoA | AVSD repair, CoA repair | 4.28 |
| 26 | AVSD | AVSD repair | 3.02 |
| 27 | Shone's complex | Repair of aortic arch, CoA, PDA ligation | 3.61 |
| 28 | Valvular/sub-PS, ASD | valvotomy, RV muscle resection | 5.98 |
| 29 | HLHS s/p Glenn | redo TCPC | 17.0 |
| 30 | TAPVR | TAPVR repair | 2.97 |
| 31 | re CoA, s/p ETE repair of CoA | re CoA repair | 7.47 |
| 32 | Unbalanced AVSD, s/p BT shunt | redo BDG | 5.31 |
| 33 | HLHS, s/p Norwood, BDG | redo TCPC | 15.1 |
| 34 | TA, hypo RV, TGA, CoA, VSD | Norwood, BT shunt, ASD enlargement | 3.1 |
| 35 | ASD, VSD, vascular ring | Ligation of double arch, ASD/VSD closure | 4.23 |
| 36 | AVSD | AVSD repair | 6.41 |
| 37 | pAVSD, Noonan syndrome | pAVSD repair | 5.71 |
| 38 | Levocardia, BAV, AR, s/p AVP | redo Ross/Konno surgery | 5.36 |
| 39 | ASD, VSD | ASD/VSD closure | 5.88 |
| 40 | TOF | TOF repair | 3.0 |

**Supplemental Table 1.**

ccTGA: congenitally corrected transposition of the great arteries, CoA: coarctation of the aorta, CAVC: complete atrioventricular canal, ALCAPA: anomalous left coronary artery from the pulmonary artery, MV: mitral valve, VSD: ventricular septal defect, PFO: patent foramen ovale, PDA: patent ductus arteriosus, AP: aortopulmonary, PAPVR: partial anomalous pulmonary venous return, ASD: atrial septal defect, TAPVR: total anomalous pulmonary venous return, RV: right ventricle, PA: pulmonary artery, AS: aortic stenosis, MR: mitral regurgitation, AVP: aortic valvuloplasty, DCM: diastolic cardiomyopathy, TV: tricuspid valve, MA: mitral atresia, DILV: double inlet left ventricle, L-TGA: L-looped transposition of the great arteries, PAB: pulmonary artery banding, HLHS: hypoplastic left heart syndrome, MS: mitral stenosis, AA: aortic atresia, TOF: tetralogy of Fallot, BT shunt: Blalock-Taussig shunt, BDG: bidirectional Glenn, TCPC: total cavopulmonary connection, AVSD: atrioventricular septal defect, AVV: atrioventricular valve, SV: systemic valve, PS: pulmonary stenosis, BAV: bicuspid aortic valve, MVP: mitral valve plasty, ETE: end-to-end, TA: tricuspid atresia, pAVSD: partial atrioventricular defect, AR: aortic regurgitation

**Supplemental Table 2.**

|  | Cutoff value | AUC |
| --- | --- | --- |
| Age (month) | 4.0 | 0.976 |
| BW (kg) | 5.71 | 0.977 |
| Ht (cm) | 59 | 0.962 |
| BSA (m^2^) | 0.31 | 0.981 |
| A (mm) | 132 | 0.946 |
| B (mm) | 77 | 0.971 |
| C (mm) | 57 | 0.866 |
| D (mm) | 66 | 0.922 |

AUC: area under curve, BW: body weight, Ht: height, BSA: body surface area, A: the thoracic width from left to right, B: the total heart size, C: the distance between the middle of vertebrae to apex, D: the distance from the carina to the apex, E: the distance between the sternum and middle of the vertebrae in side view
